# Morphological and morphometric studies on the axial skeleton of the sitatunga (*Tragelaphus spekii gratus*)

**DOI:** 10.1186/s40850-022-00157-2

**Published:** 2022-11-18

**Authors:** Kenechukwu Tobechukwu Onwuama, Chikera Samuel Ibe, Alhaji Zubair Jaji, Suleiman Olawoye Salami, Esther Solomon Kigir

**Affiliations:** 1 Department of Veterinary Anatomy, Faculty of Veterinary Medicine, University of Ilorin, Ilorin, Nigeria; 2 Department of Veterinary Anatomy, Faculty of Veterinary Medicine, Micheal Okpara University of Agriculture, Umudike, Nigeria

**Keywords:** Bone, Ribs, Skull, Sitatunga, Vertebrae

## Abstract

**Background:**

Anatomical features of the skeleton of wild animals contribute largely to their adaptation. A dearth of information on the skeletal anatomy of the sitatunga (*Tragelaphus spekii gratus*) necessitated this study. Two adult sitatunga carcasses weighing 54 kg and 57 kg were obtained after post-mortem examination. Bone preparation was achieved through cold water maceration protocol.

**Result:**

The tympanic bulla was elongated and massive, resulting in the rudimentary appearance of the styloid and muscular processes of the temporal bone. The lacrimal bone had a somewhat triangular presentation with the lacrimal foramen on the caudal border of the facial surface while its dorsal border formed the lateral margin of the frontal sinus. There was no observable lacrimal fossa on this facial surface of the lacrimal bone. The facial tubercle was absent. The vertebral column formula was C7 T13 L6 S4 C10-14, and the atlas dorsal median tubercle was smooth, devoid of ridges. The spinous process of the axis extended the entire arch length to hang little above the odontoid process. The thoracic spinous processes were oriented dorso-caudally from T1 to T11; spinous process of T12 was vertical, while that of T13 was oriented dorso-cranially. The length of the transverse process of L1 and L6 were the same, and smaller than the length of those of L2-L5. There was incomplete fusion of sacral spinous processes. Three dorsal and ventral sacral foramina were identified laterally on each side of the vertebrae. The ribs were 26 in number (13 pairs). The sternum was comprised of 5 sternabrae separated by intersternal cartilage. The average number of bones of the axial skeleton was 75. Morphometric in formation included the length of skull, mandible and ribs; body length of vertebrae and spinous process length and height of the vertebrae.

**Conclusion:**

This study recorded anatomical features and biometric information on axial skeletal bones of the Sitatunga (*Tragelaphus spekii gratus*) thereby providing baseline data for future biomedical, archaeological and comparative skeletal anatomical studies.

## Background

The knowledge of skeletal morphology as it relates to function in animals is essential for comparative anatomical studies, laboratory investigations, museum specimen mounting and archaeological deductions [1]. The apparent similar body conformation conferred by the skeleton on both wild and domestic animals, places a false assumption that similarities exist in the bones of the skeletons [2]. Addressing the dearth of information on the skeletal morphology of animals requires a detailed identification and description of each bone, elucidating unique features associated with it [3].

In this study, the axial skeleton of a semi-aquatic antelope, the sitatunga (*Tragelaphus spekii gratus*) has been given special attention due to its adaptation to habitations of this animal in the rain forest [4]. Initial study by Onwuama et al [2] has observed that the unique shape of the distal phalanx of the sitatunga suggested its adaptation for locomotion and survival in the swampy rainforest region. The animal is known to submerge completely underwater when threatened [5]. Its colloquial name in most African countries is “Water Kudu” because of their water habitat and their close relationship with the Kudu (*Tragelaphus strepciseros*). There are about 9 species of the genus, *Tragelaphus*. Some of them are the *Tragelaphus strepciseros* (greater kudu), *Tragelaphus angasii* (Nyala) and *Tragelaphus scriptus* (bushbuck). The ewe is easily distinguished from the bull; they have a prominent black dorsal stripe along the spine and one white marking on the throat, while the bulls acquire a second white marking on the throat and grow a prominent mane around the neck. Females also lack prominent horns.

Wild populations of this ungulate have experienced a drastic decrease, hence they are mostly available in zoos or as exotic games in hunting ranches [6]. In fact, they were once believed to be extinct in Ghana until the species was rediscovered by science in Avu Lagoon [7]. The adverse effects of increased human population and long term changes in water bodies are some of the challenges that threaten the existence of the sitatunga in Africa. To avoid extinction, the African Wildlife Foundation is currently establishing linkages for the establishment of more game reserves for the sitatunga [5]. In line with this, there is need to document the skeletal morphology of this ruminant as a reference for future use. Also, being a ruminant and a member of the family *Bovidae* [8], the need for an in-depth investigation into its skeletal structure to differentiate it from other studied ungulates like sheep, goat and cattle has become pertinent. A report on the anatomy of the bones of the appendicular skeleton is available in extant literature [2]. Unfortunately, attention has not been given to the anatomy of the axial skeleton of the sitatunga. To add to the reduced osteology database currently available on the sitatunga, this study investigated and documented the axial skeletal morphology of the sitatunga, thereby establishing baseline data which will help in future scientific and evolutionary studies. The objectives were to provide general, distinguishing features, measurements and number of bones making up the axial skeleton.

## Material and methods

### Animals

Two (2) female adult sitatunga carcasses weighing 54 kg and 57 kg were obtained at different times after post-mortem examination, from the Department of Veterinary Pathology, Faculty of Veterinary Medicine, University of Ilorin, Nigeria. Results of the post-mortem examinations did not indicate pathological conditions on the skeleton.

### Extraction of the axial skeleton

The bones of the axial skeleton were prepared by cold maceration as described by *Onwuama *et al [9] at the Department of Veterinary Anatomy of the same institution. The carcasses were dissected using scalpel blade to carefully remove the skin, muscles and visceral organs, leaving the bones with minimal muscular and ligament attachments. They were then put in a large container with enough water to submerge the bones. The container was covered airtight and placed under the sun. Water was changed weekly before being drained and bones recovered after one (1) month (31 days). The recovered bones were then degreased using sodium bicarbonate and sun-dried. Various segments of the axial skeleton were glued for presentation and photography which was taken using a digital camera (Nikon Coolpix 24 megapixel). The bones were studied grossly to describe their presentations and specific features according to nomenclature from Nomina Anatomica Veterinaria 2017. Measurements of the length of the skull, mandible and ribs; body length of vertebrae and spinous process length and height of the vertebrae were taken with their means ± SEM determined using Graph pad prism version 5.0.

## Results

The total average number of bones seen in the axial skeleton of this species was 75 (Table 1). This comprised bones of the skull, mandible, sternum, ribs, cervical, thoracic, lumbar, sacral and caudal vertebrae. The morphometric characteristics of the axial skeletal bones of the Sitatunga are presented in Table 2.

**Table 1:** Number of bones of the axial skeleton of the sitatunga (*Tragelaphus spekii gratus*)

Bones	Number
Skull	1
Mandible	1
Sternum	5
Ribs	26 (13 pairs)
Cervical vertebra	7
Thoracic vertebra	13
Lumbar vertebra	6
Sacral vertebra	4
Caudal vertebra	10–14
**Total average:**	**73–77 (Av. 75)**

**Table 2:** Morphometric characteristics of the axial skeletal bones of the Sitatunga (*Tragelaphus spekii gratus*)

Bone Dimension (cm)	Mean ± SEM	Bone Dimension (cm)	Mean ± SEM	Bone Dimension (cm)	Mean ± SEM
Skull length	26.05 ± 0.75	6^th^ th.v, b. length	2.250 ± 0.25	4^th^ l.v, b. length	3.000 ± 0.30
Mandible length	18.95 ± 0.35	6^th^ th.v, s.p. height	6.700 ± 0.50	4^th^ l.v, s.p. length	2.200 ± 0.10
Atlas b. length	2.250 ± 0.25	7^th^ th.v, b. length	2.250 ± 0.25	4^th^ l.v, t.p. length	3.650 ± 0.15
Atlas t.p length	4.350 ± 0.25	7^th^ th.v, s.p. height	6.300 ± 0.50	5^th^ l.v, b. length	3.050 ± 0.25
Axis b. length	5.500 ± 0.50	8^th^ th.v, b. length	2.300 ± 0.30	5^th^ l.v, s.p. length	2.100 ± 0.10
Axis s.p. length	4.050 ± 0.25	8^th^ th.v, s.p. height	5.850 ± 0.45	5^th^ l.v, t.p. length	3.950 ± 0.25
Axis s.p. height	1.050 ± 0.15	9^th^ th.v, b. length	2.300 ± 0.30	6^th^ l.v, b. length	2.850 ± 0.15
3^rd^ c.v, b. length	4.900 ± 0.50	9^th^ th.v, s.p. height	5.500 ± 0.50	6^th^ l.v, s.p. length	1.850 ± 0.05
3^rd^ c.v, s.p. height	0.400 ± 0.10	10^th^ th.v, b. length	2.300 ± 0.30	6^th^ l.v, t.p. length	3.550 ± 0.35
4^th^ c.v, b. length	4.950 ± 0.45	10^th^ th.v, s.p. height	4.800 ± 0.45	Sacrum length	9.300 ± 0.40
4^th^ c.v, s.p. height	0.500 ± 0.10	11^th^ th.v, b. length	2.350 ± 0.35	Ca. v. length	22.15 ± 0.45
5^th^ c.v, b. length	5.000 ± 0.60	11^th^ th.v, s.p. height	3.900 ± 0.40	Sternum length	13.05 ± 0.15
5^th^ c.v, s.p. height	0.750 ± 0.05	12^th^ th.v, b. length	2.450 ± 0.25	1^st^ rib length	7.950 ± 0.45
6^th^ c.v, b. length	4.050 ± 0.35	12^th^ th.v, s.p. height	2.400 ± 0.10	2^nd^ rib length	10.40 ± 0.50
6^th^ c.v, s.p. height	1.150 ± 0.05	13^th^ th.v, b. length	2.600 ± 0.20	3^rd^ rib length	13.15 ± 0.45
7^th^ c.v, b. length	3.100 ± 0.50	13^th^ th.v, s.p. height	2.000 ± 0.10	4^th^ rib length	14.40 ± 0.50
7^th^ c.v, s.p. height	2.050 ± 0.25	1^st^ l.v, b. length	2.700 ± 0.20	5^th^ rib length	19.00 ± 0.50
1^st^ th.v, b. length	2.300 ± 0.20	1^st^ l.v, s.p. length	1.950 ± 0.05	6^th^ rib length	19.70 ± 0.50
1^st^ th.v, s.p. height	4.500 ± 0.70	1^st^ l.v, t.p. length	1.250 ± 0.25	7^th^ rib length	21.85 ± 0.35
2^nd^ th.v, b. length	2.250 ± 0.25	2^nd^ l.v, b. length	2.900 ± 0.20	8^th^ rib length	23.20 ± 0.50
2^nd^ th.v, s.p. height	6.300 ± 0.90	2^nd^ l.v, s.p. length	2.000 ± 0.00	9^th^ rib length	23.05 ± 0.45
3^rd^ th.v, b. length	2.200 ± 0.30	2^nd^ l.v, t.p. length	2.600 ± 0.10	10^th^ rib length	21.00 ± 0.50
3^rd^ th.v, s.p. height	6.400 ± 0.60	3^rd^ l.v, b. length	2.950 ± 0.35	11^th^ rib length	20.10 ± 0.60
4^th^ th.v, b. length	2.200 ± 0.20	3^rd^ l.v, s.p. length	2.100 ± 0.10	12^th^ rib length	17.50 ± 0.50
4^th^ th.v, s.p. height	6.450 ± 0.25	3^rd^ l.v, t.p. length	3.250 ± 0.15	13^th^ rib length	17.05 ± 0.55
5^th^ th.v, b. length	2.250 ± 0.25				
5^th^ th.v, s.p. height	6.750 ± 0.45				

*Key:* *b* body, *t.p* transverse process, *s.p* spinous process, *c.v* cervical vertebra, *th.v* thoracic vertebra, *l.v* Lumbar vertebra, *ca.v* caudal vertebra

### The skull

**Fig. 1:**
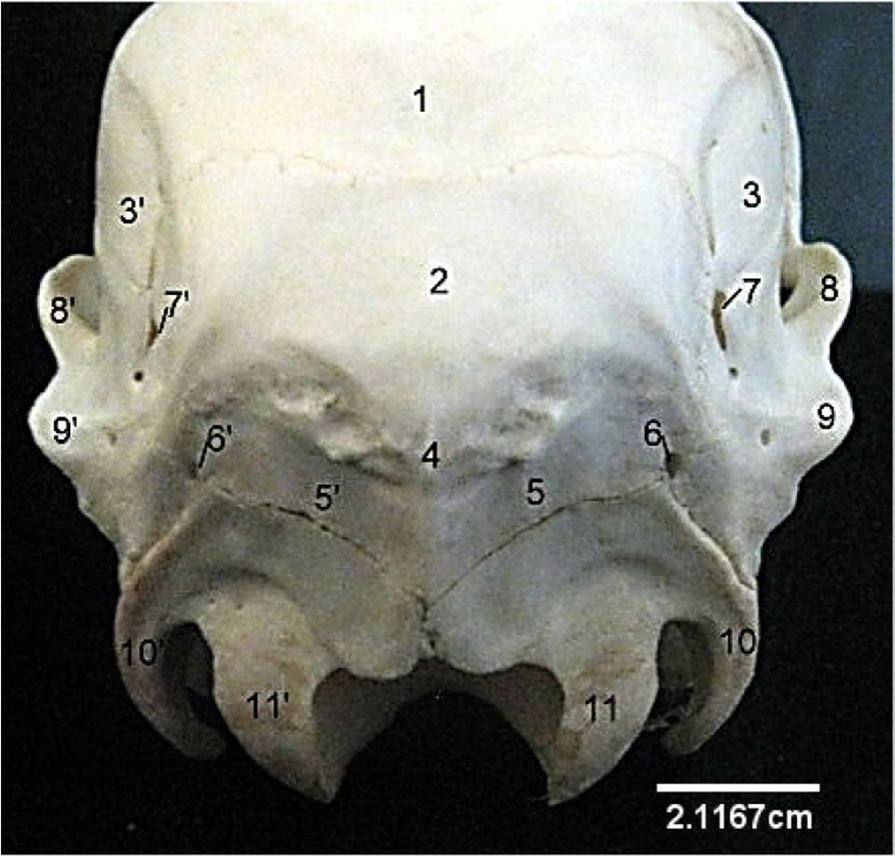
Sitatunga skull (Caudal view). 1, 3, Parietal; 2, Occipital; 4, Occipital protuberance; 5, Squamous occipital; 6, 7, Temporal canal; 8, 9, Squamous temporal; 10, Paramastoid process; 11, Occipital condyle

**Fig. 2:**
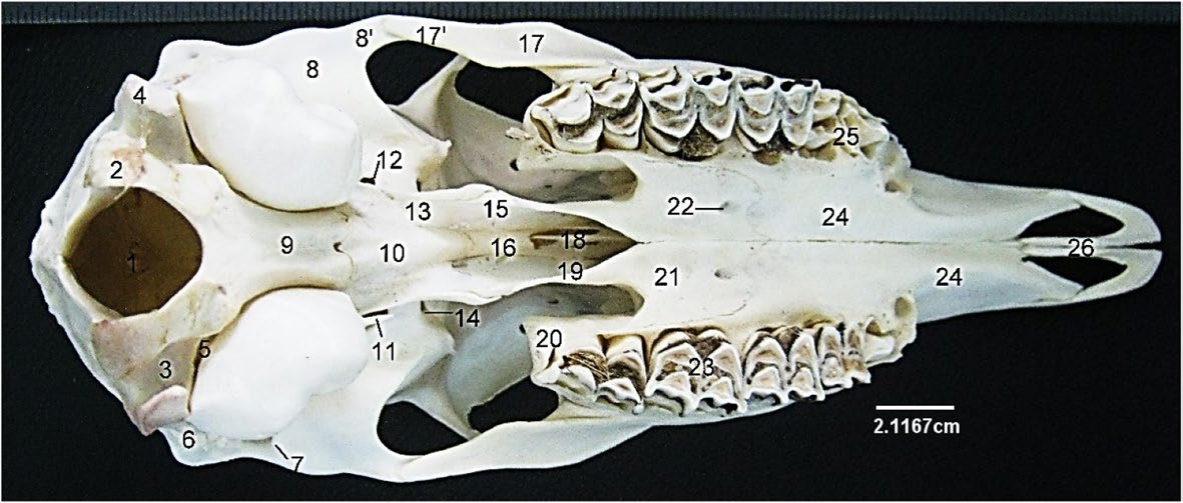
Sitatunga skull (Ventral view). 1, Foramen magnum; 2, Occipital condyle; 3, Condyloid fossa; 4, Paracondyloid process; 5, Jugular foramen; 6, Mastoid process; 7, External acoustic meatus; 8, Temporal; 8’, Zygomatic process of temporal; 9, Basilar part of occipital; 10, Basisphenoid; 11, Muscular process in front of large tympanic bulla; 12, Foramen orbitorotundum; 13, Wing of sphenoid; 14, Oval foramen; 15, Pterygoid; 16, Presphenoid; 17, Malar; 17’, Zygomatic process of malar; 18, Vomer; 19, Vertical part of palatine; 20, Maxillary tuberosity; 21, Horizontal plate of palatine; 22, Palatine foramen; 23, Molars; 24, Palatine process of maxilla; 25, Premolars; 26, Palatine process of premaxilla

**Fig. 3:**
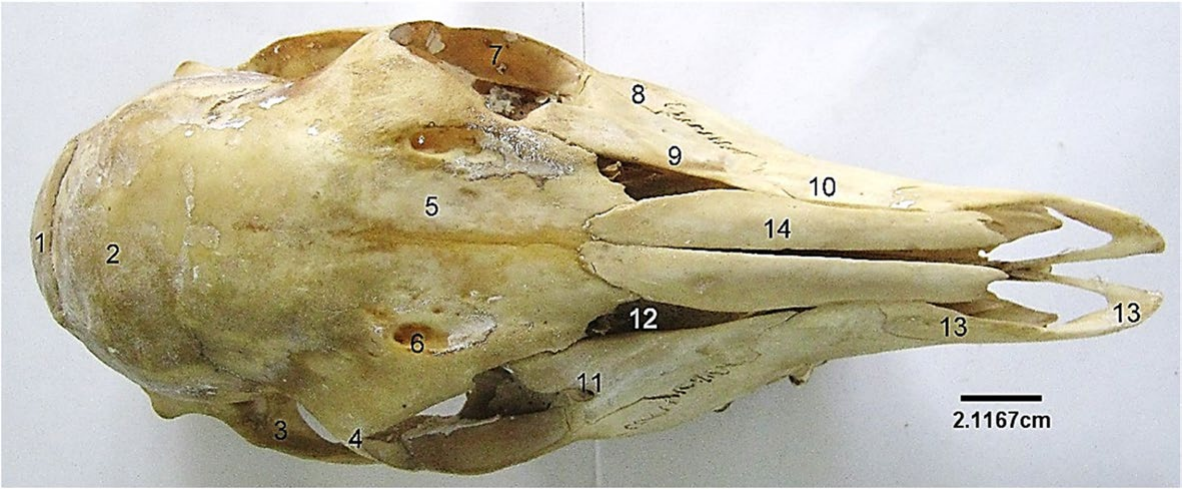
Sitatunga skull (dorsal view). 1, Occipital, 2, Parietal; 3, Temporal; 4, Orbital process of frontal; 5, Frontal; 6, Supraorbital foramen; 7, Zygomatic process of malar; 8, Malar or Zygomatic; 9, Lacrimal; 10, Maxilla; 11, Lacrimal foramen; 12, Triangular sinus; 13, Premaxilla; 14, Nasal

This presented an elongated structure made up of caudally located cranial bones and rostrally located facial bones. The bones were flat and joined together by sutures. Caudally, the cranial cavity was bounded by an unpaired occipital bone that presented a dorsal quadrilateral squamous part, lateral parts and a ventral basilar part (Fig. 1). The quadrilateral squamous part presented a convex external surface roughened at its centre, the cranial border that joined the parietal bone cranially and lateral borders that joined the lateral parts of occipital. The lateral parts were formed by the lateral paramastoid process and medial condyle. They were separated by a deep condyloid fossa that lodged two foramina medially. The ventral basilar part met cranially forming a synchondrosis with the basisphenoid (Fig. 2). These three parts of the occipital bone formed the boundaries of the large foramen magnum (Fig. 1). Dorsocaudally, a single parietal bone formed the middle part of the roof of the cranial cavity. It extended laterally and rostrally to articulate with the squamous part of temporal bone and paired frontal bones respectively (Fig. 3). The interparietal bone was absent. The paired frontal bone (separated by the interfrontal suture) formed the dorso-rostral part of the cranial cavity and the major part of the orbital wall. Each frontal bone extended a zygomatic process caudo-laterally that articulated with that of the malar (Fig. 3). They also bore the supraorbital foramen dorsally and articulated rostrally with the paired nasal bones (via the fronto-nasal suture) and lacrimal bones (via the fronto-lacrimal suture) (Fig. 3).

**Fig. 4:**
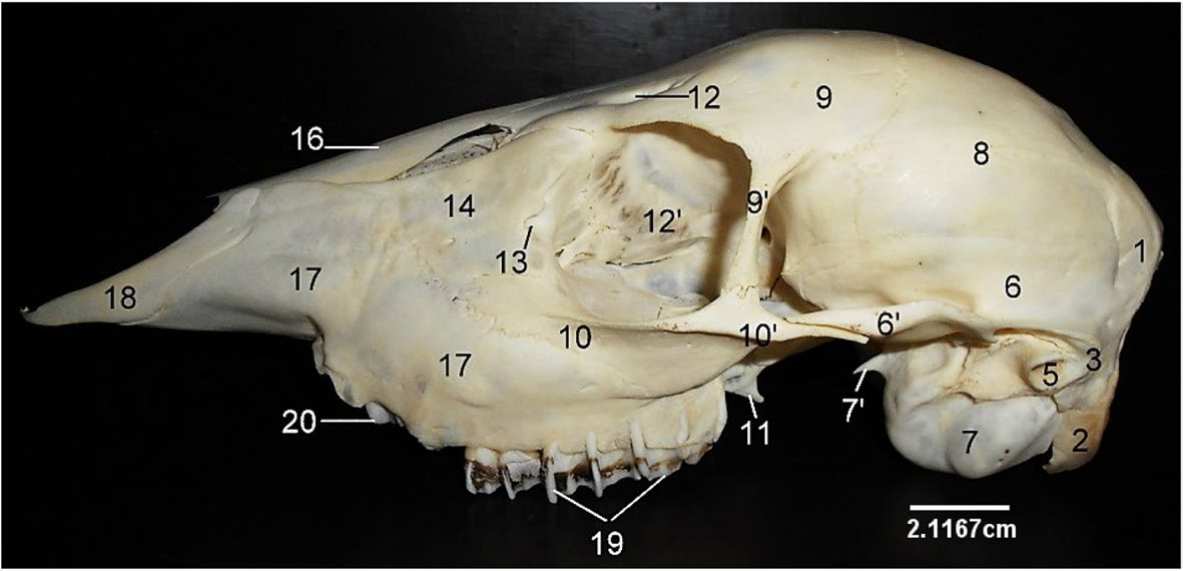
Sitatunga skull (lateral view). 1, Occipital; 2, Paramastoid process; 3, Mastoid process; 5, External acoustic meatus; 6, Squamous part of temporal; 6’, Zygomatic process of temporal; 7, Tympanic bulla; 7’, Muscular process; 8, Parietal; 9, Frontal; 10, Malar or Zygomatic10’, Zygomatic process of malar; 11, Hamulus of pterygoid; 12, Supraorbital foramen; 12’, Orbit; 13, Lacrimal foramen; 14, Lacrimal; 16, Nasal; 17, Maxilla; 18, Premaxilla; 19, Molars; 20, Premolars

The paired temporal bone formed the ventro-lateral boundaries of the cranium composed of a petrous and squamous part (Fig. 2). The squamous part presented a temporal fossa dorsally and an articular surface ventrally which extends a rostral zygomatic process to the malar. It also presented a caudo–lateral temporal canal caudal to the articular surface for mandibular condyle articulation (Fig. 4). The petrous part was ventrally located and composed of a medial massive tympanic bulla, styloid process and mastoid process (Fig. 2). Between the basilar part of occipital and the tympanic bulla was the position of the rostral lacerated foramen and the caudal jugular foramen (Fig. 2).

The sphenoid bone (Fig. 2) formed part of the ventral surface of the cranial cavity. It continued rostral to the occipital (basilar part) to present a rostral part (pre-sphenoid), a caudal part (basiphenoid), medial vertical wings and lateral horizontal wings (which bore the cranial and caudal large orbitorotundum and oval foramina respectively). The optic foramen opened cranial to the orbitorotundum foramen. The vomer was placed cranial to the presphenoid projecting rostro-medially into the floor of the nasal cavity (Fig. 2).

The ethmoid bone was placed cranial to the cranium projecting the crista galli medio-caudally, perpendicular plate medio-cranially and the cribriform plate laterally. The palatine bone, also located ventrally was composed of a vertical and horizontal part. The vertical part formed the boundary of the posterior choanae while the horizontal part bore the palatine foramen. Placed between the vertical wing of the sphenoid and vertical palatine was the pterygoid bone, a small flat thin quadrilateral bone ending ventrally in a hamulus.

The nasal bone (Fig. 4) was rostral to the frontal bone articulated to it, via the fronto-nasal suture. Rostrally, its ventrolateral border joins with the dorsal border of the maxilla and premaxilla. The paired lacrimal bone formed the caudolateral aspect of the nasal cavity and rostral margin of the orbit (Fig. 4). It presented a triangular outline and articulated rostro-ventrally with the maxilla and malar bone. Its caudal border presented a lacrimal foramen and a bulla further into the orbital cavity. The frontal, nasal and lacrimal bones formed the caudal (serrated), dorsal and ventral borders respectively of a triangular shaped sinus that exposed the dorsal nasal concha.

The malar/zygomatic bone was located on the ventral part of the orbit and lateral to the lacrimal. It projected a caudal zygomatic process and a dorsal orbital process that met with the zygomatic process of the temporal and orbital process of the frontal respectively. Its ventral border articulated with the dorsocaudal border of the maxilla (Fig. 4).

The maxilla (Fig. 4) was located latero-ventrally and presented at its ventral margin, alveolar processes and alveoli for dental insertions. Its ventral palatine process formed the rostral part of the hard palate while its dorsal border articulated with the malar, lacrimal and nasal bones via the malo-maxillary, lacrimo-maxillary and naso-maxillary sutures respectively. Rostroventrally to the first premolar, the maxilla presented two (large and small) infraorbital foramina (Fig. 4).

**Fig. 5:**
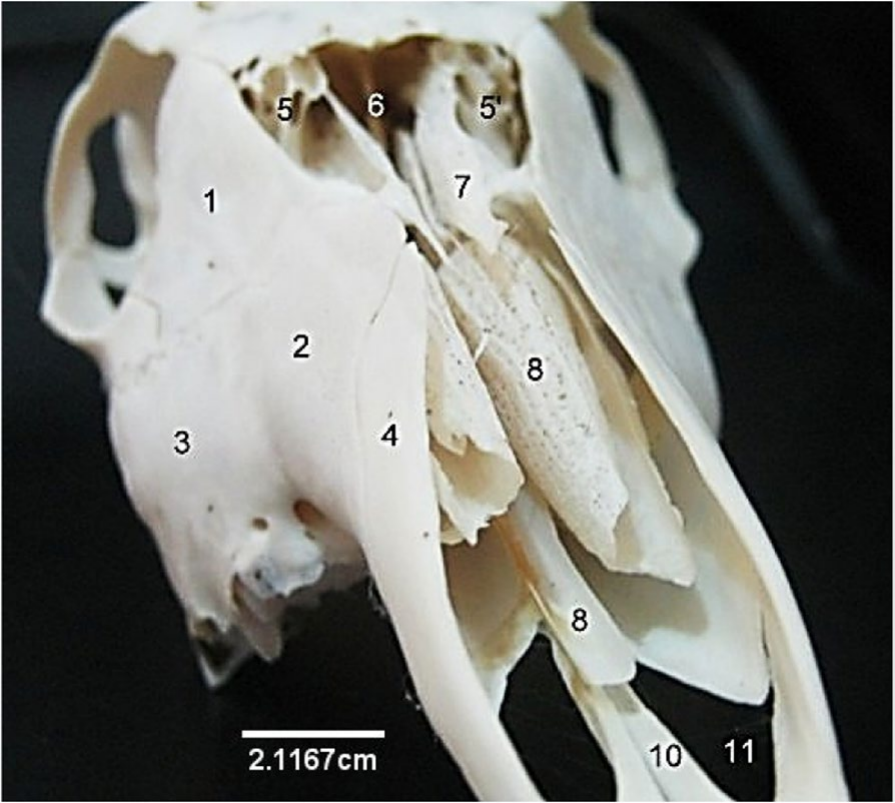
Sitatunga skull (cranial view). 1, Lacrimal; 2, 3, Maxilla; 4, Premaxilla; 5, Sinus; 6, Perpendicular plate; 7, Ethmoturbinate; top 8, Dorsal turbinate; below 8, Vomer; 10, Palatine process of premaxilla; 11, Palatine fissure

The premaxilla was an irregularly shaped bone articulating with the rostral border of the maxilla. It is composed of a body laterally, a nasal process dorsally and a palatine process ventrally on which the palatine fissure was located (Fig. 3). The three scroll-like (dorsal, middle and ventral) turbinate bones were located on the medial aspect of the maxilla and composed of numerous fenestrations (Fig. 5).

**Fig. 6:**
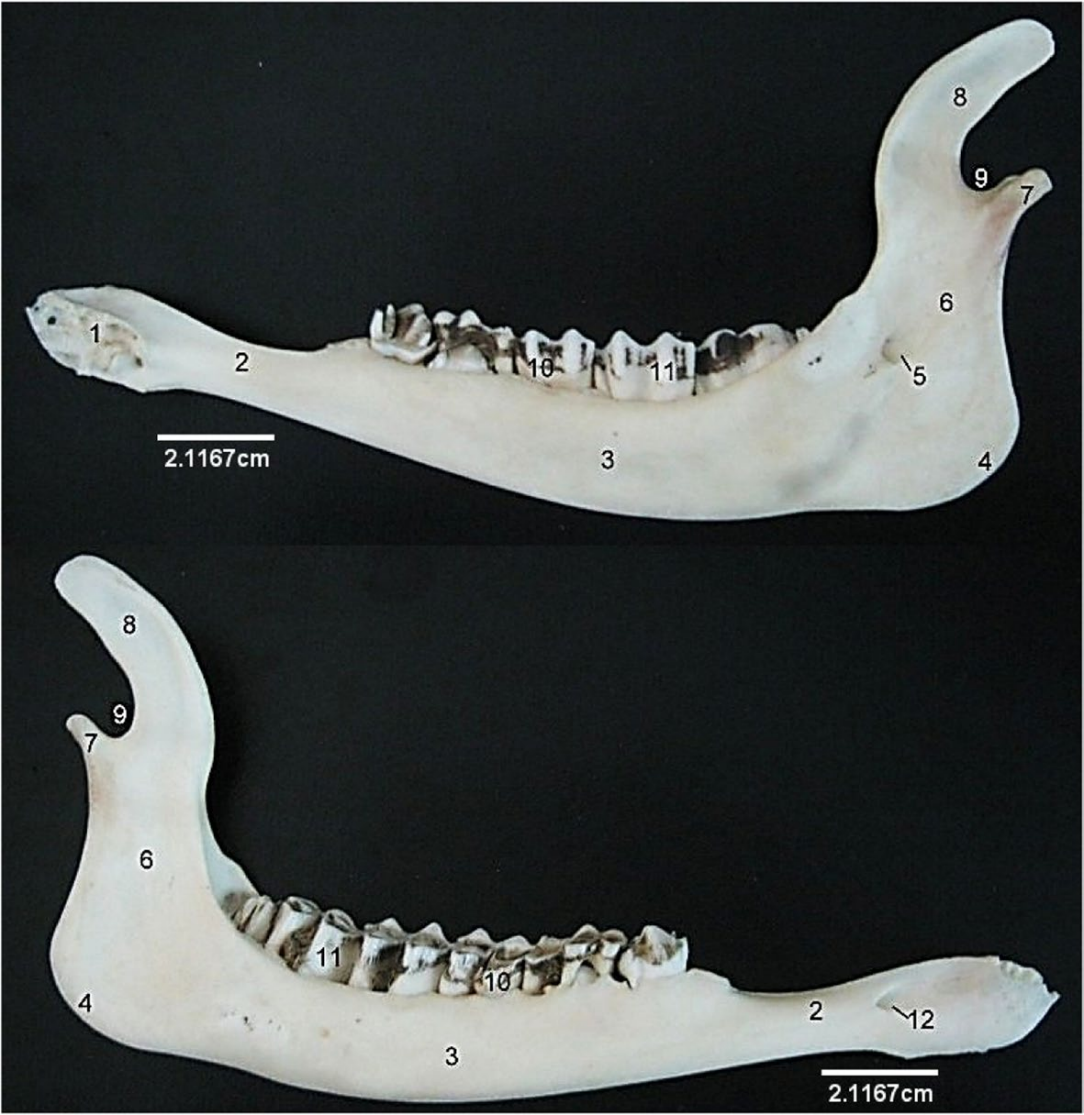
Sitatunga mandible (Medial (**A**) and lateral (**B**) views). 1, Mandibular symphysis; 2, Diastema; 3, Horizontal ramus; 4, Mandibular angle; 5, Mandibular foramen; 6, Vertical ramus; 7, Mandibular condyle; 8, Coronoid process; 9, Mandibular notch; 10, Premolars; 11, Molars, 12, Mental foramen

The mandible (Fig. 6) presented a v-shaped appearance having two halves joined rostrally to form a symphysis. The body located rostrally, presented dorsal alveoli for insertion of the incisors while bearing a lateral mental foramen. The horizontal rami located caudal to the body presented alveoli on their dorsal margin for insertion of the molars and premolars. The diastema, a space between the incisors and the first premolar was bounded ventrally by the dorsal mandibular crest. The vertical rami projected a curved coronoid process, rostrally and a slightly bent mandibular condyle, caudally. These were separated by a sigmoid/mandibular notch. The medial aspect of this rami bore the mandibular foramen.

### The Vertebral Column

This comprised 7 cervical, 13 thoracic, 6 lumbar, 4 sacral and 10–14 caudal vertebrae, giving a formula of C7, T13, L6, S4, C10-14. They all consisted of a body with an arch constructed dorsally to enclose the median vertebral foramen. Emanating from these were the spinous processes dorso-medially, articular processes dorso-laterally and transverse processes laterally. Some specific vertebrae bore foramina, and other processes (mammillary and ventral).

**Fig. 7:**
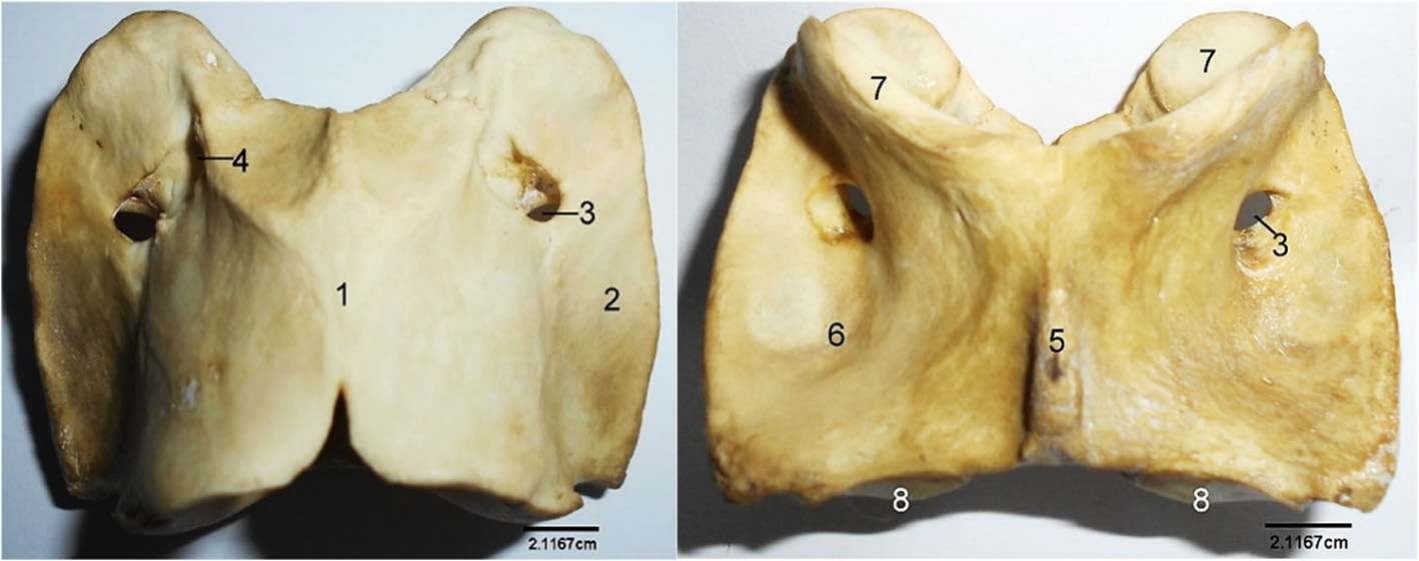
Sitatunga Atlas (Dorsal and ventral views). 1, Dorsal tubercle; 2, Wing; 3, Alar foramen; 4, Intervertebral foramen; 5, Ventral tubercle; 6, Atlanta fossa; 7, Cranial articular surface; 8, Caudal articular surface

The cervical vertebrae formed the skeletal framework of the neck. The first cervical vertebra, the atlas (Fig. 7) presented an almost non-existent body forming a strong ring (that enclosed the vertebral foramen) from which a pair of modified and undivided transverse process, the wings projected laterally. Convex and concave articular surfaces of the body featured cranially and caudally, respectively. It also presented dorsal and ventral median tubercles. The dorsal median tubercle was devoid of ridges. Each wing presented a convex dorsal surface and a concave ventral depression, the atlanta fossa. Its cranial aspect presented an inner lateral vertebral foramen and an outer alar foramen.

**Fig. 8:**
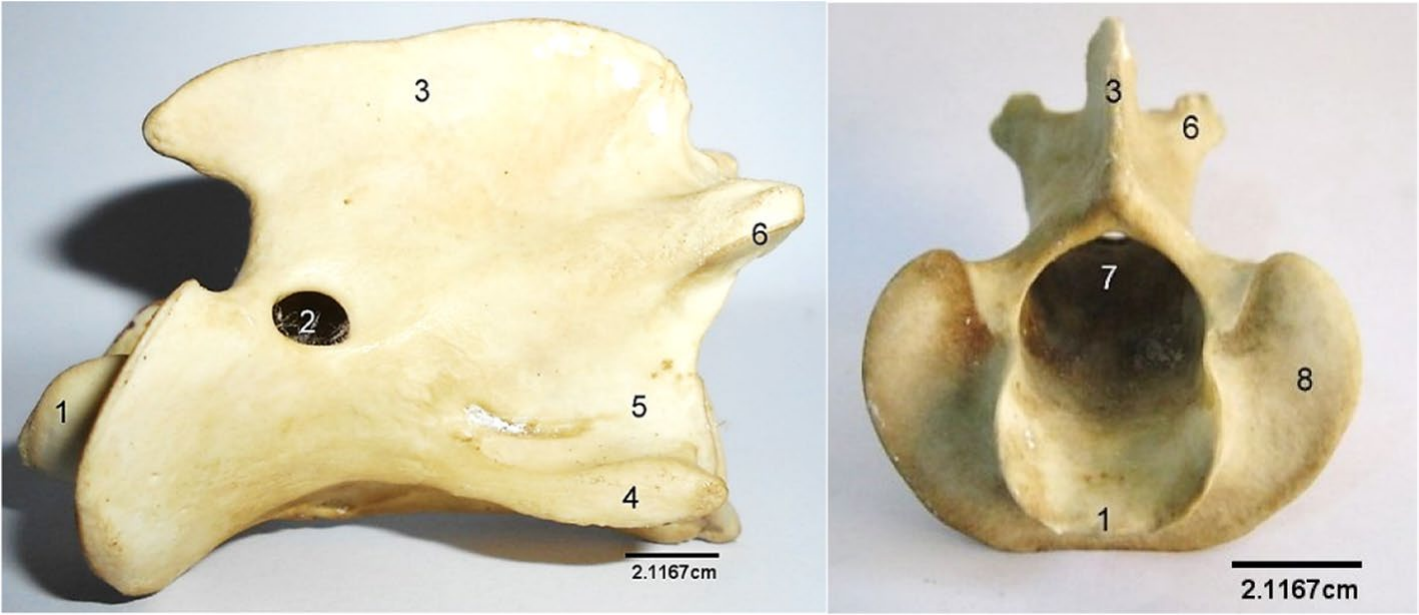
Sitatunga Axis (Lateral and cranial views). 1, Odontoid process; 2, Lateral vertebral foramen; 3, Spinous process; 4, Transverse process; 5, Body; 6, Caudal articular process; 7, Vertebral foramen; 8, Cranial articular surface

The second cervical vertebra, the axis (Fig. 8), projected craniomedially, the odontoid process having a dorsal concave and ventral convex surfaces. The lateral vertebral foramen perforated the arches to open into the large medial vertebral foramen. The caudal articular process of the axis projected dorso-laterally from the arch while the transverse process projected only caudo-laterally. The spinous process ran dorsally on the length of the arch forming a crest that hung over the odontoid process.

**Fig. 9:**
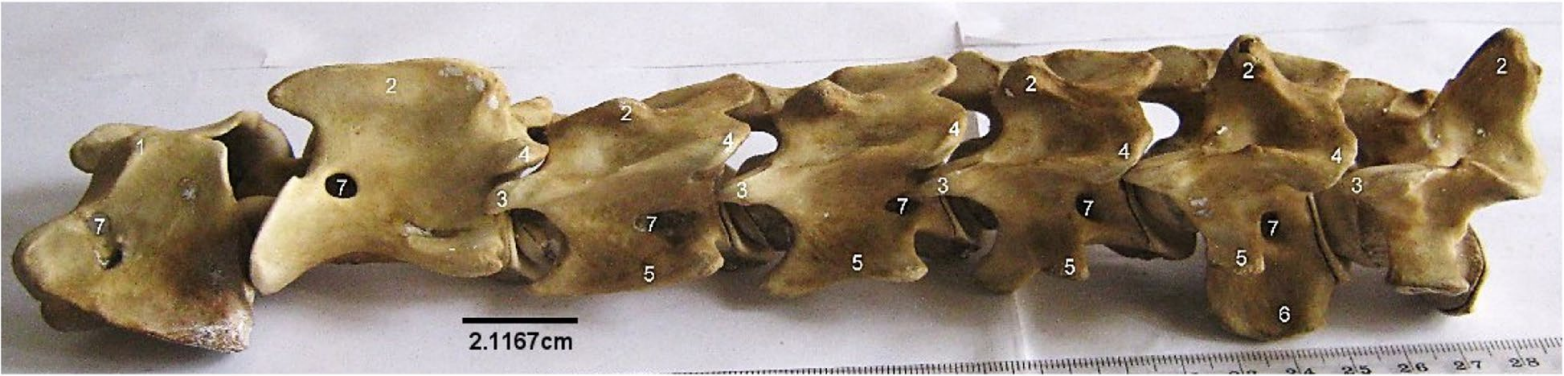
Sitatunga cervical vertebrae (Lateral view). 1, dorsal tubercle; 2, Spinous process; 3, Cranial articular process; 4, Caudal articular process; 5, Transverse process; 6, Ventral process; 7, Intervertebral foramina

The 3rd to 6th cervical vertebrae (Fig. 9) presented the typical configuration of a vertebra in addition to a transverse foramen placed laterally in between the transverse process and the arch parallel to the body. Their spinous processes increased caudally. The 5th and 6th cervical vertebrae presented a ventral process each, that projected cranially only in the 5th. The 7th cervical vertebra appeared like the thoracic vertebrae as it possessed the longest spinous process than the 3rd to 6th, caudal coastal facet and a rudimentary transverse foramen that was only visible cranially (Fig. 9).

**Fig. 10:**
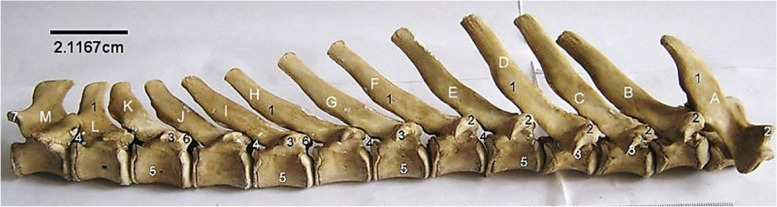
Sitatunga thoracic vertebrae (lateral view). A-M, 1st to 13th thoracic vertebrae; 1, Spinous process; 2, Mammillary process; 3, Transverse process; 4, Intervertebral foramen; 5, Body; 6, Cranial articular process; 7, Caudal articular process

**Fig. 11:**
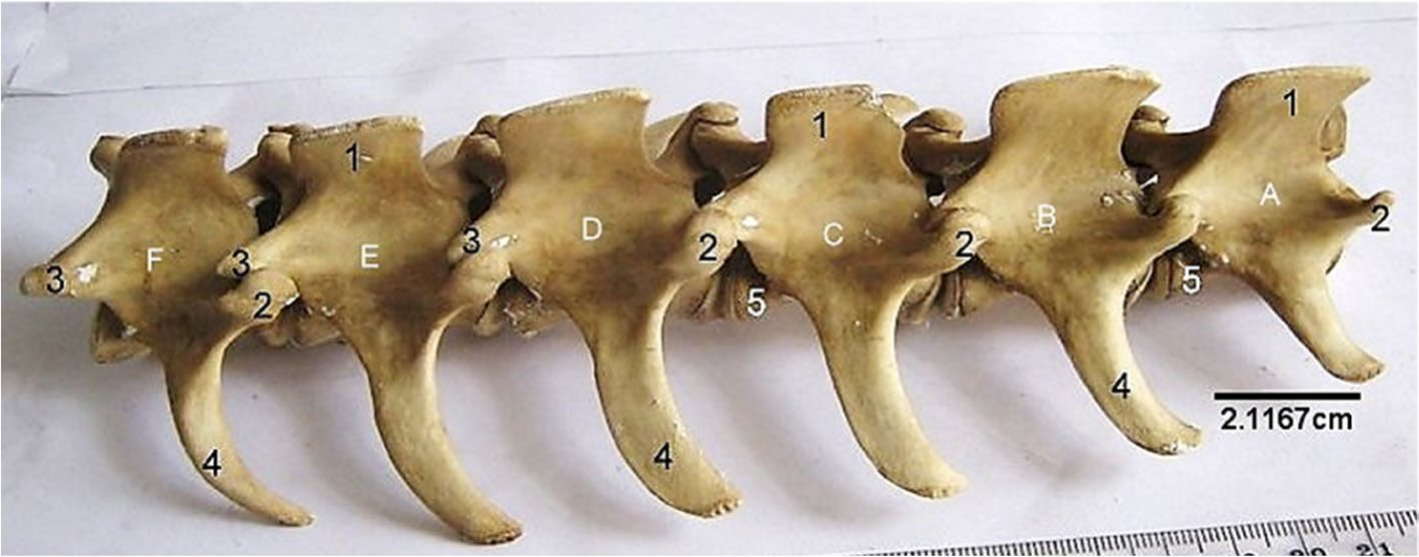
Sitatunga lumbar vertebrae (Lateral view). A-F, 1st to 6th lumbar vertebrae; 1, Spinous process; 2, Cranial articular process; 3, Caudal articular process; 4, Transverse process; 5, Body

The thoracic vertebrae (Fig. 10) formed the dorsum of the thoracic cavity presenting well developed bodies with convex cranial and concave caudal surfaces both bearing costal facets laterally for head of rib articulation. However, the last thoracic vertebra lacked a caudal costal facet. The spinous processes increased from T1 to T4, reaching maximum height in the T4. From here, the spinous processes decreased caudally. They were oriented dorso-caudally from T1 to T11. Spinous process of T12 was vertical, while that of T13 was oriented dorso-cranially. These spinous processes increased in length caudally, peaking at T4 before diminishing towards the last. Mammillary processes (between the cranial articular process and transverse process) were present on T1 to T7 (Fig. 11). The cranial and caudal articular processes were not as prominent as those of the cervical vertebrae.

The lumbar vertebrae (Fig. 11) formed the dorsum of the abdomen presenting, in addition to the features of a typical vertebra, long dorso-ventrally flattened transverse processes that curved cranially and lacked the transverse foramen. The length of the transverse process of L1 and L6 were the same, and smaller than the length of those of L2-L5. The cranial articular processes had concave facets that articulated with convex articular facet of the caudal articular process of the preceding vertebrae. Their spinous processes were short, broad, and ended sharply as a crest (Fig. 11).

**Fig. 12:**
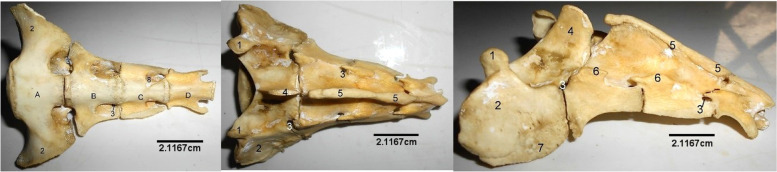
Sitatunga Sacrum (Ventral, dorsal and lateral views). 1, Cranial articular process; 2, Wing; 3, Dorsal sacral foramina; 4, Spinous process; 5, Dorsal sacral crest; 6, Interrupted lateral sacral crest; 7, Auricular surface; 8, Ventral Sacral foramina. A-D, Sacral vertebra

The sacrum (Fig. 12) was composed of 4 fused bones with the first being the largest and featuring similar processes like the lumbar vertebra. Their spinous processes fused to form a median sacral crest. Three dorsal and ventral sacral foramina were identified laterally on each side of the vertebrae.

**Fig. 13:**
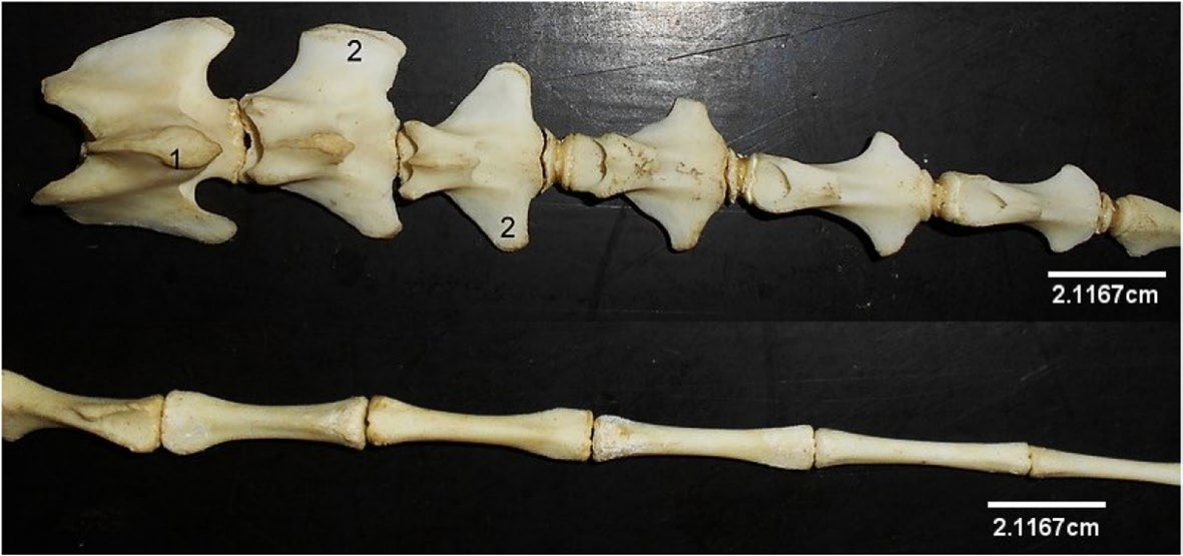
Sitatunga Caudal vertebrae (dorsal view). 1, Spinous process; 2, Transverse process

The caudal vertebrae (Fig. 13) presented a cranial portion having the typical vertebral features that diminished gradually through the series. Only the bodies of the last few caudal vertebrae were evident.

**Fig. 14:**
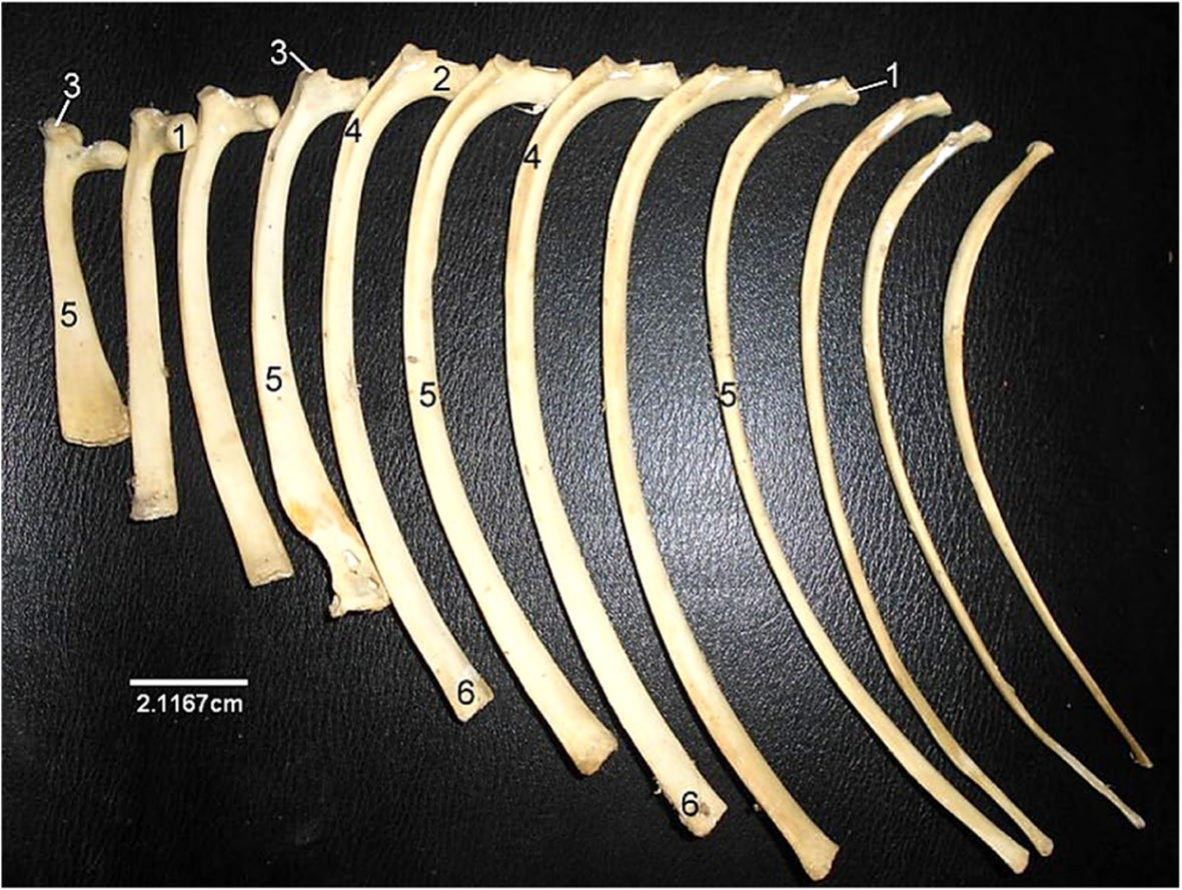
Sitatunga Ribs (lateral view). 1, Head; 2, Neck; 3, Tubercle; 4, Groove; 5, Shaft; 6, Distal extremity

The ribs (Fig. 14) were 26 in number (13 pairs). The dorsal parts were bony while the ventral parts were cartilaginous presenting surfaces for attachment to the sternum. The proximal extremity of each rib was made up of a cranially located head and neck, and a caudally located tubercle. The cranial border of the shaft was concave while its caudal border was convex in appearance. The lateral and medial surfaces were lined by the coastal groove.

**Fig. 15:**
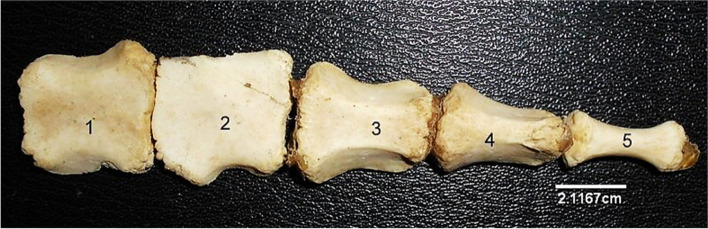
Sitatunga Sternum (Dorsal view). 1, First sternabra; 2, Second sternabra; 3, Third sternabra; 4, Fourth sternabra; 5, Fifth sternabra

The Sternum (Fig. 15) presented 5 sternabrae separated by intersternal cartilage. The first sternabra presented a somewhat square shape. This shape gradually became triangular towards the fourth sternabra before a slim shape at the 5^th^ sternabra with visible body and extremities. Some parts of the Xyphoid cartilage was seen attached to the caudal extremity.

## Discussion

The Axial skeleton of the sitatunga (*Tragelaphus spekii gratus*) presented morphological features that were comparable with those of other members of the same family, *Bovidae*. The Skull of the sitatunga with a more elongated presentation than those of the sheep and goat [10], presented similar features with the exception of the following: the petrous part of the temporal bone presented a massive bulging tympanic bulla that resulted in a rudimentary appearance of the styloid and muscular processes. Given that the major parts of the middle and inner ear are located in the bulla; it is suggestive that these structures being housed are also enlarged so as to enhance its sense of hearing in the wild. The single supraorbital foramen observed in this study is similar to the observation in the sheep by Onuk et al [11]. Sarma [12] and Shawulu et al [13] reported one in certain breeds of goats, but some other breeds possessed a second supraorbital foramen. In the roe deer, an increased number, up to 4 supraorbital foramina, were reported by Onuk et al [11]. Furthermore, the oval foramen was formed by the sphenoid bone only, similar to the goat as reported by [13].

The lacrimal bone also presented a major difference from those of the sheep and goat [14, 15] in that it had a large somewhat triangular presentation with the lacrimal foramen on its caudal border while its dorsal border formed the lateral margin of the frontal sinus. Only one lacrimal foramen was observed in the present study; in the roe deer, a small ruminant of non-*Bovidae* family (it belongs to the *Cervidae* family), two lacrimal foramina were observed by [11], dorsal and ventral lacrimal foramina on the facial surface of the lacrimal bone. This implies that the skull morphology my not be dependent on feeding patterns but evolved phylogenetically in the taxonomic tree. The absence of lacrimal fossa reported in the present work has also been reported in the goat, but is present in the sheep and roe deer [11]. The facial tubercle, which was expected on the lateral aspect of the maxilla as seen in the sheep and goat was absent in this species which is also a unique feature of this animal. Onuk et al [11] did not observe this facial tuber in the roe deer.

In the present study, a mental foramen was identified on the lateral surface of the mandible. Conversely, Shawulu et al [13] observed double openings, indicating second mental foramina in some of the goats studied, an attributed it to the existence of an accessary mental nerve in the affected goats. Also, while a functional mandibular symphysis was observed in this study, Shawulu et al [13] reported that the mandible of some goats does not join at the mandibular symphysis, creating an independent rotatory movement of the mandibles during feeding in such goats.

The vertebral column presented a formula of C7 T13 L6 S4 C10-14 similar to what was reported in other small ruminants of the same *Bovidae* family such as the goat and sheep [14, 16] except the caudal vertebrae that varied in number (Table 3). Conversely, the cattle, also a ruminant of the *Bovidae* family, has an extra sacral vertebra [17], while the giraffe, a ruminant of non-*Bovidae* family (it belongs to the *Giraffidae* family) has 14 thoracic, 5 lumbar and 3 sacral vertebrae [17]. This implies that mammalian vertebral column formula is neither dependent on taxonomy nor on feeding behaviour. The morphology of each vertebra conformed to the normal architecture of body, arch and process.

**Table 3:** Comparative anatomic features between Sitatunga and domestic ruminants axial skeletal bones [18]

Anatomical features	Sitatunga	Goat	Sheep	Cattle
Interparietal bone	absent	absent	present	present
Supraorbital groove	absent	absent	absent	present
Triangular sinus	large	medium	small	absent
Facial tubercle	absent	Present	present	present
Tympanic bulla	massive	massive	massive	small
Muscular and styloid process	rudimentary	rudimentary	rudimentary	prominent
Mandibular coronoid process	curves caudally	curves caudally	curves caudally	projects erect
Vertebral formula	C_7_T_13_L_6_S_4_Ca_10 – 14_	C_7_T_13_L_6 – 7_S_4_Ca_16 – 18_	C_7_T_13_L_6 – 7_S_4_Ca_16 – 18_	C_7_T_13_L_6_ S_5_Ca_18 – 20_

The dorsal median tubercle of the atlas lacked a conspicuous ridge, and none of the paired transverse processes was divided. In another ruminant, the barking deer (*Muntiacus muntjak*), Suri et al [19] reported that the dorsal median tubercle was characterised by a prominent ridge, and each of the wings (transverse processes) was divided into a cranio-ventral and caudo-dorsal processes. The sixth cervical vertebra bore the ventral process, the 1st to 7th thoracic bore the mammillary process while the accessory process was absent. The above findings were similar to what was reported in literatures on the sheep [10] and goat [14].

The highest point of the thoracic vertebrae, measured by the highest spinous process, differ in ruminants. In the present study, T4 was the highest point. The highest point was T5 in the barking deer [19], giraffe [17], gazelle [20] and T7 on white spotted deer (*Axis axis*) [21]. The orientation of the spinous processes of the entire thoracic vertebrae observed in this study is similar to that of the gazelles [20] and cattle [17], both of the same *Bovidae* family with the sitatunga. It is also similar with that of the giraffe [17], another ruminant, but of the *Giraffidae* family. This implies that this orientation of the thoracic spinous processes of ruminants is not a phylogenetic evolutionary trait.

The cranial curvature of the lumbar transverse processes observed in the sitatunga from this study was also observed in the barking deer [19], the blackbuck antelope [22] and the gazelles [20]. The dorsoventrally flattened nature of each of the lumbar transverse processes reported here has also been reported in the barking deer by [19]. Choudhary et al [22] described it as a long plate of bone in the blackbuck. This indicates the dorso-ventral flattened nature of the bone in this species. The variation in the length of the transverse process of the lumbar vertebra observed in the present study is different from that of the blackbuck antelope; [22] reported that the length of the first transverse process was the shortest, which gradually increased up to the last.

The number of sacral vertebrae and sacral foramina vary among ruminants of the same *Bovidae* family. For example, the blackbuck (also called Indian antelope) and the gazelle, both being ruminants of the *Bovidae* family, have extra sacral vertebrae each, as reported by Choudhary et al [22] and Yilmaz et al [20], respectively. The number is also 5 in the barking deer [19] and the white spotted deer [21], both being ruminants of the non-*Bovidae* family. While three pairs of sacral foramina were each observed on the dorsal and ventral sacral surfaces in the present study, four were observed in the blackbuck [22] and in the gazelle [20]. Suri et al [19] reported two dorsal and four ventral sacral foramina in the barking deer. The first sacral vertebra was not involved in the fusion of the spinous processes to form the median sacral crest, in this study. This has also been reported in the Karaman sheep [23] and Blackbuck [22]. On the contrary, this is different from that of the mountain sheep and gazelle in which Taşbaş et al [23] and Yilmaz et al [20], respectively, reported that spinous processes of all the sacral vertebrae fused to form the median sacral crest. The loss of typical structure of vertebra in the caudal vertebra after the first few caudal vertebrae that was observed in the present study is similar to other ruminants previously studied.

## Conclusion

This study on the gross anatomy of the axial skeleton of the sitatunga (*Tragelaphus spekii gratus)* has been able to elucidate morphological features on bones making up this region of the skeleton while pointing out differences where visible as it concerns members of its family.

## Data Availability

The datasets used or analyzed during the current study are available from the corresponding author on reasonable request.
